# Cyclosporine-A-induced nephrotoxicity in children with minimal-change nephrotic syndrome: long-term treatment up to 10 years

**DOI:** 10.1007/s00467-007-0709-6

**Published:** 2008-04-01

**Authors:** Birgitta Kranz, Udo Vester, Rainer Büscher, Anne-Margret Wingen, Peter F. Hoyer

**Affiliations:** Clinic of Pediatric Nephrology, University Clinic Essen, Hufelandstrasse 55, 45122 Essen, Germany

**Keywords:** Minimal-change nephrotic syndrome, Cyclosporine A, Long-term outcome, Nephrotoxicity, Glomerular filtration rate

## Abstract

The impact of cyclosporine A (CsA) therapy in patients with steroid-dependent nephrotic-syndrome (SDNS) on long-term renal function is controversial. Data beyond 5 years are rare. Long-term renal function was evaluated in children with SDNS with and without CsA therapy, especially beyond 5 years. Twenty children were treated with CsA (study group) for a mean of 5.4 ± 2.2 years (ten patients for 5–11 years). Glomerular filtration rate (GFR) was calculated before and after 3 and 12 months and at latest follow-up of therapy. Fifteen children with cyclophosphamide-treated SDNS without CsA served as controls. In the study group, GFR decreased within 12 months from 136 ± 19 to 120 ± 31, to 114 ± 14 ml/min per 1.73 m^2^ at latest follow-up (*p* < 0.0001). Patients with CsA > 5 years had a GFR of 111 ± 14 ml/min per 1.73 m^2^ at latest follow-up without a GFR below 90 ml/min per 1.73 m^2^. No CsA toxicity was found in biopsies. In the control group, GFR dropped within 3 months, from 137 ± 27 to 130 ± 24, to 126 ± 19 ml/min per 1.73 m^2^ at latest follow-up (*p* = 0.1). Patients with and without nephrotoxic CsA therapy showed a drop in GFR. In CsA-treated patients, GFR was about 12% lower at latest follow-up compared with patients without nephrotoxic therapy but always remained within normal range. CsA seems to be safe, even in long-term treatment for more than 5 years.

## Introduction

Minimal-change nephrotic syndrome (MCNS) in children is characterised by steroid responsiveness, subsequent relapses and a benign prognosis concerning renal function [1]. Patients with steroid-dependent nephrotic syndrome (SDNS) and frequent relapsing nephrotic syndrome (FRNS) with steroid-toxic side effects are recommended for treatment with cyclophosphamide (CP). The overall rate of cumulative sustained remission after therapy with CP, however, was only 24% after 10 years [2]. The efficacy of cyclosporine A (CsA) in the treatment of steroid-sensitive nephrotic syndrome (SSNS) has been well demonstrated [3–8]. Early withdraw of CsA leads to relapses of the SDNS. So patients remain dependent on CsA for years. CsA was identified as being nephrotoxic, inducing tubulointerstitial fibrosis [9], vasoconstriction leading to reduced renal plasma flow and glomerular filtration rate (GFR) [10]. The potential risk of long-term CsA therapy to induce chronic renal failure in patients with MCNS has been discussed controversially. Seikaly et al. [11] found no drop in GFR but a histological progression of tubulointerstitial lesions in patients treated with CsA in comparison with those without CsA. Hulton et al. [5, 12] demonstrated a significant reduction of GFR within the first 3 months after introduction of CsA. Thereafter, the GFR remained stable. In contrast, Inoue et al. [6] could not detect a significant impairment of renal function in patients with CsA therapy. The interpretation of several studies focussing on the impact of CsA therapy on long-term renal function is limited and controversial.

1. Most authors investigate the influence of CsA on renal function in nephrotic children with different histologic entities, not considering their different risk to progress to chronic renal failure. Thus Niaudet et al., Habib and Niaudet, and Gregory et al. [7, 13, 14] did not differentiate between MCNS and focal segmental glomerulosclerosis (FSGS) in their studies evaluating long-term renal function in nephrotic children with CsA therapy.
2. The physiological drop in GFR after temporary hyperfiltration during a relapse often has been neglected. Relapse-associated hypoproteinemia and hypoalbuminaemia lead to oedema and increased GFR [15]. Hulton et al. [12] considered that concomitant therapy with steroids could contribute to an increase of GFR due to the mineralocorticoid effect. Consequently, steroid cessation could lead to a physiological drop in GFR.
3. Several studies claim to address the long-term effect of CsA therapy on renal function, but actually, many of them do not extend more than 2–3 years [5, 6, 7, 12, 16], depicting the short-term effect of CsA on renal function.
4. No study evaluated an additional control group without nephrotoxic therapy to demonstrate the physiological development of GFR in patients with SDNS.

In this study, we focus on the long-term renal function in 20 children with biopsy-proven MCNS treated with CsA for a mean of 5.36 ± 2.2 (range 2–11) years. The development of GFR in these patients is compared with a control group with SDNS without CsA therapy.

## Patients and methods

Between 1993 and 2004, 20 children (eight girls and 12 boys) with biopsy-proven MCNS were treated with CsA for a mean of 5.4 ± 2.2 (median 5.0, range 2.1–11.0) years. Patients only qualified for this retrospective analysis after a minimum CsA therapy duration of 2 years. The definitions and criteria for NS, remission, relapse and steroid dependency were those used by the International Study of Kidney Disease in Children (ISKDC) and the Arbeitsgemeinschaft Pädiatrische Nephrolgie (APN) [17, 18].

All patients had SDNS and were treated with cyclophosphamide (CP) prior to CsA without achieving sustained remission. CsA treatment was started with a dosage of 100–150 mg/m^2^ per body surface area (BSA) in two divided doses. CsA dose was adjusted to a target blood level of 80–120 ng/ml. Side effects such as hypertension, hypertrichosis and gingival hyperplasia were not consistently documented and could not be considered in this study. Renal function (GFR) was calculated by the Schwartz formula ([19], κ-factor 0.55) before and after 3 and 12 months and at the latest follow-up of CsA therapy. Renal biopsy under CsA therapy was performed in five patients after 4.9–7.0 years. All patients starting CsA therapy were still on a standard relapse treatment with prednisone (40 mg/m^2^ every 48 h) that was discontinued after 4 weeks. At the reviews after 3 and 12 months and latest follow-up, all patients were in remission and had no steroid therapy.

In the control group, 15 children (seven girls, eight boys) with SDNS treated with CP were evaluated for long-term renal function. GFR was calculated before and after therapy with CP and at the latest follow-up (mean 4.9 ± 3.4 years). According to APN recommendations, these patients were treated with CP with 2 mg/kg body weight for 12 weeks [18, 20]. As concomitant medication, all patients in the control group received prednisone 60 mg/m^2^ every 48 h tapered to 10 mg/m^2^ every 48 h and discontinued when CP was stopped. At latest follow-up, all patients were in remission without further steroid therapy.

### Statistical analysis

Statistical analysis was performed employing SPSS 12.0 for windows. Data were expressed as mean ± standard deviation. Student’s *t* test was performed, and a *p* value <0.05 was regarded as significant.

## Results

**Fig. 1:**
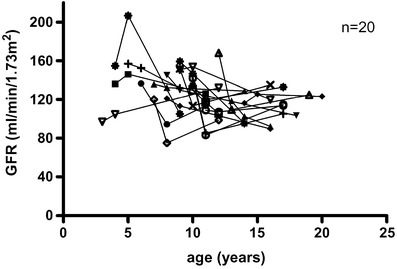
Development of the glomerular filtration rate (GFR) of all patients on cyclosporin A (CsA) therapy (*n* = 20)

**Fig. 2:**
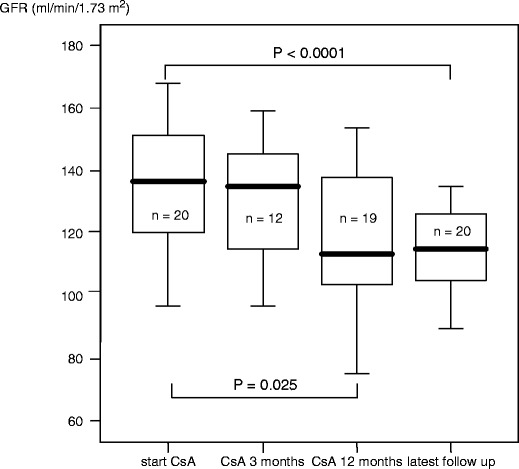
Boxplot demonstrating the development of glomerular filtration rate (GFR) in patients on cyclosporin A (CsA) therapy (*n* = 20)

Twenty paediatric patients with SDNS were treated with CsA due to failing CP therapy for a mean of 5.4 ± 2.2  (median 5.0, range 2.1–11.0) years. The mean starting dosage was 130 ± 32 mg/m^2^ BSA per day. The mean CsA dosage after 12 months was 126 ± 26 mg/m^2^ BSA per day, with a trough level of 99 ± 27 ng/ml. The mean age at the beginning of CsA therapy was 8.4 ± 3.0  (median 8.6, range 3.1–14.2) years. GFR calculated at the beginning, at months 3 and 12 and at the latest follow-up of CsA treatment was 136 ± 19 (median 136), 130 ± 20 (median 135), 120 ± 31 (median 113) and 114 ± 14 (median 114) ml/min per 1.73 m^2^, respectively (Table 1 and Fig. 1). Unfortunately, data of only 12 patients were available for the calculation of GFR at month 3. The decline in GFR from the beginning to month 12 and to the latest follow-up was significant, with *p* = 0.025 and *p* < 0.0001, respectively (Fig. 2). The drop in GFR within the first 12 months was 11.8% and during the whole follow-up 14.9%. Three patients reestablished their GFR after cessation of CsA. No patient had a GFR below 90 ml/min per 1.73 m^2^, and 17 patients had a GFR above 105 ml/min per 1.73 m^2^ at latest follow-up. CsA trough level at latest follow-up was 85 ± 62 ng/ml; mean CsA dosage was 105 ± 34 mg/m^2^ BSA per day.

**Table 1:** Patient characteristics [mean ± standard deviation (SD)] and renal function calculated by glomerular filtration rate (GFR in ml/min per 1.73 m^2^; mean ± SD) in patients with cyclosporine A (CsA, study group) and with cyclophosphamide (CP, control group)

	Study group *n* = 20		Control group *n* = 15	
Age at start of MCNS (years)	4.4 ± 2.2		4.0 ± 2.9	
Age at start of CP (years)	5.7 ± 2.2		6.0 ± 3.2	
Age at start of CsA (years)	8.4 ± 3.0		–	
CsA trough level at month 12 (ng/ml)	98.8 ± 26.9		–	
Follow-up time (years)	5.4 ± 2.2		4.9 ± 3.4	
GFR at start of CP	138.7 ± 24.6	*p* = 0.4	137.7 ± 27.6	*p* = 0.3
	Median 132		Median 131.6	
	Range 119–181		Range 95–196	
GFR at stop of CP	130 ± 30.9		130.3 ± 24.7	
	Median 135.9		Median 131.1	
	Range 82–164		Range 93–178	
GFR at start of CsA	136.3 ± 19.0	*p*<0.0001		
	Median 136.5			
	Range 97–186			
GFR at latest follow-up	114.5 ± 14.5		126.4 ± 19.8	*p* = 0.1
	Median 114.2		Median 124.6	
	Range 89–135		Range 92–156	

*MCNS* minimal-change nephrotic syndrome, *GFR* glomerular filtration rate

CsA was effective in 19 of 20 patients, leading to long-term remission or reduction of relapses to an infrequent relapsing NS (IRNS). Chlorambucil was introduced in one patient due to frequent relapses after CsA cessation. In three patients, arterial hypertension was treated; in one patient, antihypertensive therapy could be stopped after CsA cessation. Ten patients were treated with CsA for more than 5 (median 6.5, mean 7.0 ± 1.8,  range 5.0–11.0) years. GFR at latest follow-up was 111 ± 14  (median 109, range 90–132) ml/min per 1.73 m^2^. Five patients underwent renal biopsy after 5.1–7.3 years after CsA therapy had been started before they were transferred to adult nephrologists. CsA-associated nephrotoxicity was not reported by an experienced renal pathologist (Prof. U. Helmchen, Hamburg, Germany). CsA was stopped in nine patients (mean age 13.1 ± 2.5 years) after a mean of 4.9 ± 1.9 (median 4.4, range 2.3–9.0) years due to long-term sustained remission in all but one patient. GFR remained normal after CsA cessation, with 120 ± 25 (median 116) and 123 ± 10 (median 125, range 105.18–134.98) ml/min per 1.73 m^2^ at time of therapy cessation and at latest follow-up, respectively.

**Fig. 3:**
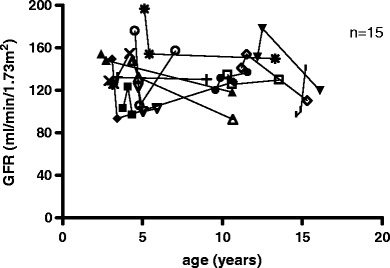
Development of glomerular filtration rate (GFR) of patients without cyclosporin A (CsA) therapy (*n* = 15)

**Fig. 4:**
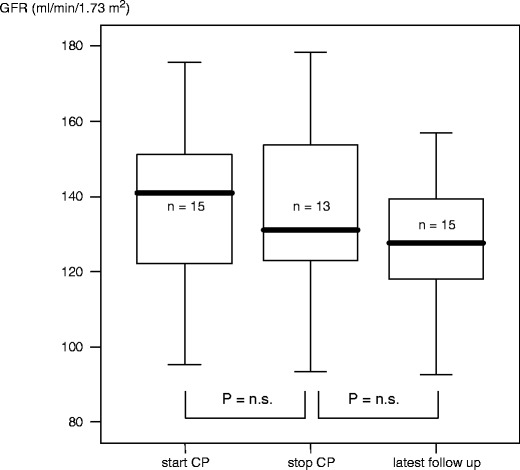
Boxplot demonstrating the development of glomerular filtration rate (GFR) in patients without cyclosporin A (CsA) therapy (*n* = 15)

The control group consisted of 15 patients with SDNS who were treated with CP at a mean age of 6.0 ± 3.2 (median 4.6) years. At the start of CP, GFR was 138 ± 28 (median 132) ml/min per 1.73 m^2^. After 3 months, GFR dropped to 130 ± 25 ml (median 131) ml/min per 1.73 m^2^ (*p* = 0.3). GFR at latest follow-up (mean follow-up 4.9 ± 3.4 years) was 126 ± 20 (median 125) ml/min per 1.73 m^2^ (*p* = 0.1, Figs. 3 and 4). This drop was in the order of 8% but not statistically significant.

Table 1 summarises the development of GFR in both patient groups, starting with the GFR before CP therapy. Patients of both groups had an initial decline in GFR during CP therapy.

## Discussion

Histologically, the nephrotoxic effect of CsA has been defined by Mihatch et al. [9]: most frequent lesions attributable to CsA are tubular atrophy, interstitial fibrosis and arteriolar hyalinosis. However, a review of the literature demonstrates that it is difficult to predict the influence of CsA on long-term renal function in patients with MCNS. Some authors did not find a significant reduction in renal function under CsA therapy in nephrotic children [4, 8] and only minor changes in renal histology without progression of CsA tubulointerstitial lesions [14, 21]. Others emphasised reduced GFR [5, 12], reduced renal plasma flow [22] or progression of tubulointerstitial lesions histologically [11, 23] in patients with NS and CsA therapy. Histology progression could not be associated with decrease in renal function [11, 23] or duration of CsA therapy [7]. Myers et al. [24] postulated that in case of striped interstitial fibrosis, some nephrons undergo atrophy while their function could be compensated for by unaffected nephrons. This may mask nephrotoxicity by measurements of normal GFR. In summary, a clear proven association between impaired renal function and long-term CsA therapy in patients with MCNS could not be found. But hints permit the question regarding the safety of long-term CsA therapy in children with a benign renal disease.

Comparison of results of this study with established opinions (e.g. [8, 12, 23]) is difficult:

1. Most studies mix different aetiological entities with different potential to progress to chronic renal failure (MCNS vs. SRNS and FSGS; [4, 7, 13, 16, 21, 22]). The impairment of renal function in patients with FSGS and other types of SRNS cannot reliably be distinguished from CsA nephrotoxicity, as those entities have a high risk for chronic renal failure on their own.
2. In most studies, the follow-up period is restricted to 2–3 years; in single studies up to 5 years. In this study, at least ten patients underwent long-term CsA therapy > 5 years. No other study investigated patients with such a long treatment period [5–8, 11–14, 23].

Neuhaus et al. and Ganesan et al. [8, 23] evaluated patients with MCNS only and an extended follow-up time of 5 years. They evaluated nine patients, with one being identified with FSGS as the underlying disease in the follow-up biopsy. All patients received CsA with higher trough levels and higher CsA dosage (level: mean of 220 ng/ml, range 141–270 ng/ml, CsA dosage 126 ± 29 mg/m^2^ per day) than accepted for our study group. The method for CsA level measurements was a polyclonal assay resulting in higher levels compared with monoclonal assays. Nevertheless, the CsA levels and dosage were still higher than in our study group. CsA toxicity was proven in 3/9 patients histologically. Ganesan et al. [23] found histological signs for CsA toxicity in 79% and renal insufficiency with a GFR < 80 in 4/19 patients without correlation to histological changes. Again, that study mixed patients with steroid-resistant and steroid-responsive NS, not distinguishing between the different clinical entities.

In our study, only patients with histologically proven MCNS were evaluated for long-term renal function under CsA therapy. Patients qualified for the study only after a minimum treatment duration of 2 years. The follow-up period extended over a mean of 5.4 years, with ten patients who had been treated between 5 and 11 years. At least in half of the patients with long-term CsA therapy > 5 years, CsA toxicity was excluded by biopsy. Also in our study group, the GFR of patients with CsA therapy remained within the normal range, with a drop within the first 12 months (mean drop 11.8%) and remaining stable thereafter, with a mean GFR of 115 ± 16 ml/min per 1.73 m^2^. No patient progressed to chronic renal failure, even after therapy duration of more than 10 years. Hulton et al. [5, 12] showed comparable results for patients with CsA therapy, with an initial drop in GFR and a constant renal function afterwards. But the study is restricted to a short follow-up of 2 years in four patients only. In our study, a significant reincrease in GFR after therapy cessation was not found. In general, these patients showed excellent renal function in the long-term follow-up (mean 123 ml/min per 1.73 m^2^). In contrast, patients in Hulton et al.’s studies showed a worse GFR of 93 that reincreased back to a GFR > 100 ml/min per 1.73 m^2^ after CsA had been stopped.

Interestingly, in our control group of patients without CsA therapy, an initial drop in GFR was also found. Those patients showed a mean drop in GFR of about 5% within the 12 weeks of CP therapy, and GFR even dropped about 8% until the latest follow-up. Probably, the observed decrease in GFR in patients with CsA therapy cannot only be attributed to the hitherto described nephrotoxic effect of CsA.

Hulton et al. [12] mentioned the potential increase of GFR caused by the mineralocorticoid effect of concomitant steroid medication. One could speculate that the cessation of steroids might lead to a physiological drop in GFR. Additionally, a possible but not proven interpretation should be discussed: Part of the GFR reduction may be attributable to normalisation of hyperfiltration in nephrotic children during the first months after initiation of immunosuppressive therapy. In patients with relapsing nephrotic syndrome, long-term immunosuppressive drugs (e.g. cyclophosphamide, CsA, chlorambucil) are initiated after urine remission has been achieved; complete remission of serum albumin normally is not awaited.

Relapse-associated hypoproteinemia and hypoalbuminemia lead to oedema and an increase in GFR. This is explained by the decrease of the colloid osmotic pressure in the glomerular capillary. Reduced total serum protein leads to decreased colloid osmotic pressure and consequently to increased GFR [15]. Normalisation of the increased filtration extends over weeks and leads to a decrease in GFR physiologically. This phenomenon may explain the drop in GFR, even in patients without nephrotoxic treatment.

In summary, this study offers long-term follow-up of 20 patients with proven SSNS treated with CsA for a median of 5 years. Initially, the patients showed a drop in GFR but remained stable afterwards. None developed chronic renal failure. Nevertheless, the generalisation of these results are limited, as the study was retrospective, and only 5/20 patients underwent renal biopsy under long-term nephrotoxic treatment. It is questionable whether a prospective study to evaluate the outcome of long-term CsA therapy in children with MCNS is reasonable in the future. Newer, nonnephrotoxic drugs, e.g. mycophenolate mofetil (MMF) are of concern and need to be evaluated for efficacy and safety in the treatment of SDNS and FRNS. However, our analysis demonstrates that even long-term therapy with CsA for more than 5 years in children with MCNS is safe and does not impair renal function.

## Abbreviations

SDNS: steroid-dependent nephrotic syndrome
IRNS: infrequent relapsing nephrotic syndrome
FRNS: frequent relapsing nephrotic syndrome
SRNS: steroid-resistant nephrotic syndrome
SSNS: steroid-sensitive nephrotic syndrome
FSGS: focal segmental glomerulosclerosis
MCNS: minimal-change nephrotic syndrome
CsA: cyclosporine A
CP: cyclophosphamide
GFR: glomerular filtration rate
